# Regulation of aPKC activity by Nup358 dependent SUMO modification

**DOI:** 10.1038/srep34100

**Published:** 2016-09-29

**Authors:** Santosh Kumar Yadav, Indrasen Magre, Aditi Singh, Deepak Khuperkar, Jomon Joseph

**Affiliations:** 1 National Centre for Cell Science, S.P. Pune University Campus, Ganeshkhind, Pune 411007, India.

## Abstract

Atypical PKC (aPKC) family members are involved in regulation of diverse cellular processes, including cell polarization. aPKCs are known to be activated by phosphorylation of specific threonine residues in the activation loop and turn motif. They can also be stimulated by interaction with Cdc42~GTP-Par6 complex. Here we report that PKCζ, a member of the aPKC family, is activated by SUMOylation. We show that aPKC is endogenously modified by SUMO1 and the nucleoporin Nup358 acts as its SUMO E3 ligase. Results from *in vitro* SUMOylation and kinase assays showed that the modification enhances the kinase activity of PKCζ by ~10-fold. By monitoring the phosphorylation of Lethal giant larvae (Lgl), a downstream target of aPKC, we confirmed these findings *in vivo*. Consistent with the function of Nup358 as a SUMO E3 ligase for aPKC, depletion of Nup358 attenuated the extent of SUMOylation and the activity of aPKC. Moreover, overexpression of the C-terminal fragment of Nup358 that possesses the E3 ligase activity enhanced SUMOylation of endogenous aPKC and its kinase activity. Collectively, our studies reveal a role for Nup358-dependent SUMOylation in the regulation of aPKC activity and provide a framework for understanding the role of Nup358 in cell polarity.

Development and growth of an organism depend on the ability of cells to respond to extrinsic and intrinsic stimuli through distinct signaling cascades. Kinases are shown to be the major class of proteins involved in the relay of these signals^1^. Protein kinase C (PKC) makes about 2% of all kinases found in the cell. PKCs are serine/threonine kinases that are highly conserved in eukaryotes and the number of PKCs increases with complexity of the organism; in yeast (*S. cerevisiae*) there is one PKC, in fruit fly (*D. melanogaster*) there are five, while in mammals, there are 12 PKCs^2^. PKCs are broadly classified into four subgroups on the basis of structure and function. The classical PKCs (*e.g.* PKCα, PKCβ, PKCγ) are activated by Ca^2+^ and diacylglycerol (DAG). Novel PKCs (*e.g.* PKCδ, PKCε, PKCθ, PKCη) are activated by DAG but not by Ca^2+^. Atypical PKCs (aPKCs) are neither activated by DAG nor by Ca^2+^ and comprises of PKCι/λ and PKCζ^2^. The members of PKN subfamily (PKN-1, PKN-2 and PKN-3) possess Rho-GTP binding domains, which regulate their activities^3^.

The aPKC sub-family proteins have been well known for their role in generation and maintenance of polarity by their asymmetric localization within the cell^4^. Broadly, there are three well characterized polarity complexes, namely, the Par3-Par6-aPKC complex, Scribble-Dlg-Lgl and the Crumbs-PALS complex^5^,^6^. aPKC is a component of the Par polarity complex and is central to its functionality. Downstream to Par complex, aPKC is known to phosphorylate and regulate a range of polarity proteins, namely, Lgl, Par3, Crumbs, GSK3β, MARCKS, LGN and Lin5/NuMA^7^. Phosphorylation can affect the target protein in various ways; for example, phosphorylation of Par3 by aPKC causes it to dissociate from the Par3-Par6-aPKC complex, while phosphorylation of Lgl prevents it from localizing to the apical membrane^8^. Two activation modes of aPKC involving phosphorylation are known; one by phosphoinositide-dependent kinase-1 (PDK1)-mediated phosphorylation of specific threonine residue (T410 in PKCζ and T408 in PKCλ) in the activation loop (A-loop)^9^ and the other by mTORC2-directed phosphorylation of T560 in the turn motif (TM) of PKCζ^10^. Additionally, aPKC can be activated in a spatio-temporal manner by binding with the Cdc42~GTP-Par6 complex through its PB1 domain^11^,^12^. Interestingly, it is found that during epithelial polarization and asymmetric division of neural stem cells, aPKC makes mutually exclusive complexes with Par3 and Lgl^13^,^14^,^15^. This appears to be important for defining the membrane domains required for generation and maintenance of cell polarity^8^. The members of aPKC family are also involved in other signaling pathways^3^.

SUMOylation, a post-translational modification involving covalent conjugation of SUMO with the target protein, affects the behavior of modified proteins in many ways^16^,^17^. SUMO proteins are highly conserved across different species and are shown to be involved in diverse cellular processes^17^. Four paralogs of SUMO exist in mammals, namely, SUMO1, SUMO2, SUMO3 and SUMO4. SUMO1 shares ~50% homology with SUMO2/3. SUMO2 however shows ~95% homology with SUMO3. Involvement of SUMO4 in protein conjugation is less studied. The consequence of SUMOylation could be different based on the SUMO paralog involved in the modification^16^,^17^. Poly-SUMOylation usually occurs with SUMO2/3^16^,^17^. SUMO conjugation requires three sets of enzymes; E1, the SUMO-activating enzyme (consists of SAE1 and SAE2 subunits), E2, the SUMO-conjugating enzyme (UBC9) and a group of SUMO E3 ligases^18^. SUMO E3 ligases include members of protein inhibitor of activated STAT (PIAS) family, Pc2 and the nucleoporin Nup358^17^. Few of the proteins known to be SUMOylated *in vivo* by Nup358 include topoisomerase II^19^, borealin^20^ and Ran^21^. SUMO specific proteases (SENPs) are involved in maturation of SUMO and deconjugation of the SUMO moiety from SUMOylated substrate proteins^22^.

We recently showed that Nup358 interacts with and acts upstream of aPKC in regulating cell polarity during neuronal differentiation^23^. Nup358 at its C-terminal region harbors two internal repeats (IRs), which have been shown to possess SUMO E3 ligase activity^24^,^25^. Nup358 also interacts with other components of Par polarity complex, Par3 and Par6^23^. We tested if components of Par complex could be SUMO targets. Here we show that aPKC is SUMOylated *in vivo* and *in vitro*, and Nup358 acts as the E3 ligase. Furthermore, our studies indicated that SUMOylation enhances the activity of aPKC, thus revealing an additional mode of activation of this conserved kinase.

## Results

**Figure 1:**
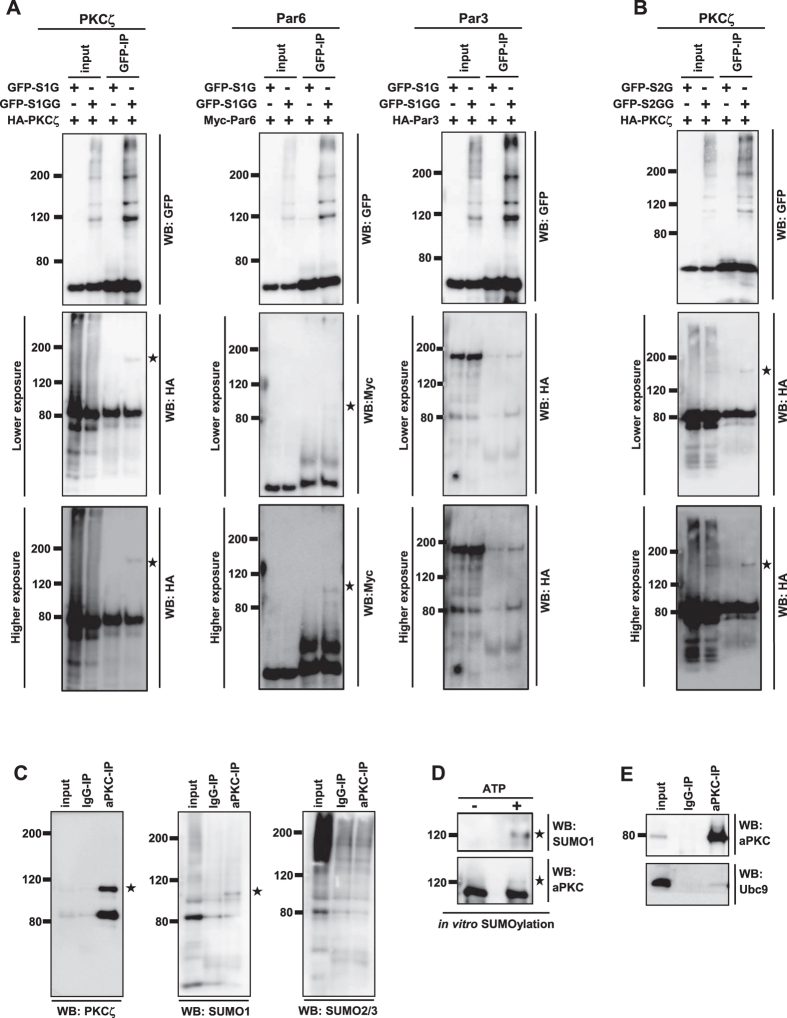
SUMOylation of aPKC. (**A**) Lysates prepared from HEK293T cells co-expressing HA-PKCζ, Myc-Par6 or HA-Par3 with GFP-SUMO1G or GFP-SUMO1GG were subjected to immunoprecipitation (IP) using GFP-specific antibodies and the immunoprecipitates were analyzed for the presence of specific proteins by western blotting (WB) using indicated antibodies. *indicates SUMO modified bands. Note that SUMOylated bands could be detected in the cases of HA-PKCζ and Myc-Par6, not in case of HA-Par3. (**B**) Cells co-expressing HA-PKCζ and GFP-SUMO2G or GFP-SUMO2GG were immunoprecipitated with GFP antibodies and probed for the presence of PKCζ using HA-specific antibodies. *indicates SUMO modified band. (**C**) HEK293T cells were lysed and IP was performed with control mouse IgG (IgG-IP) or mouse anti-aPKC (aPKC-IP) antibodies and the immunoprecipitates were subjected to western analysis using SUMO1 or SUMO2/3 antibodies. (**D**) SUMOylation of recombinant PKCζ was performed using *in vitro* SUMOylation kit as per manufacturer’s instruction. The products were analyzed by western blotting using indicated antibodies (**E**) HEK293T cells were lysed and subjected to IP using mouse IgG (IgG-IP) or mouse anti-aPKC antibodies (aPKC-IP) and western analysis was performed to monitor the presence of Ubc9.

Nup358, a well characterized SUMO E3 ligase, was shown to interact with the Par polarity complex and thereby regulate polarity in differentiating neurons^23^. To check whether any of the members of this complex is post-translationally modified by SUMO conjugation, we overexpressed GFP-tagged version of conjugatable (SUMO1GG) or non-conjugatable (SUMO1G) form of SUMO1^26^ along with the HA-Par3, Myc-Par6 and HA-PKCζ in HEK293T cells and monitored for the presence of a modified band in the GFP immunoprecipitates. We could detect high molecular weight bands for HA-PKCζ and Myc-Par6 (Fig. 1A) in cells co-transfected with SUMO1GG, but not with SUMO1G. The results suggested that PKCζ and Par6, but not Par3, were specifically modified with SUMO1 (Fig. 1A). Although both PKCζ and Par6 were found to be SUMOylated, we focused on characterizing PKCζ SUMOylation in detail. Co-expression and immunoprecipitation assays showed that PKCζ could be modified with SUMO2 as well (Fig. 1B).

To test the endogenous SUMOylation, immunoprecipitation of aPKC was performed in HEK293T lysates using specific antibodies and analyzed the immunoprecipitates for the presence of SUMO1 or SUMO 2/3. The results clearly indicated that endogenous aPKC is modified by SUMO1 (Fig. 1C). No specific SUMO2/3 positive bands were detected with aPKC immunoprecipitate, suggesting that although PKCζ gets modified by SUMO2/3 in overexpressed conditions (Fig. 1C), endogenous aPKC is preferably modified by SUMO1. SUMOylation of PKCζ was also confirmed using an *in vitro* SUMO conjugation assay (Fig. 1D). Consistent with the ability to get SUMOylated, aPKC also interacted with the SUMO E2 enzyme Ubc9 (Fig. 1E).

**Figure 2:**
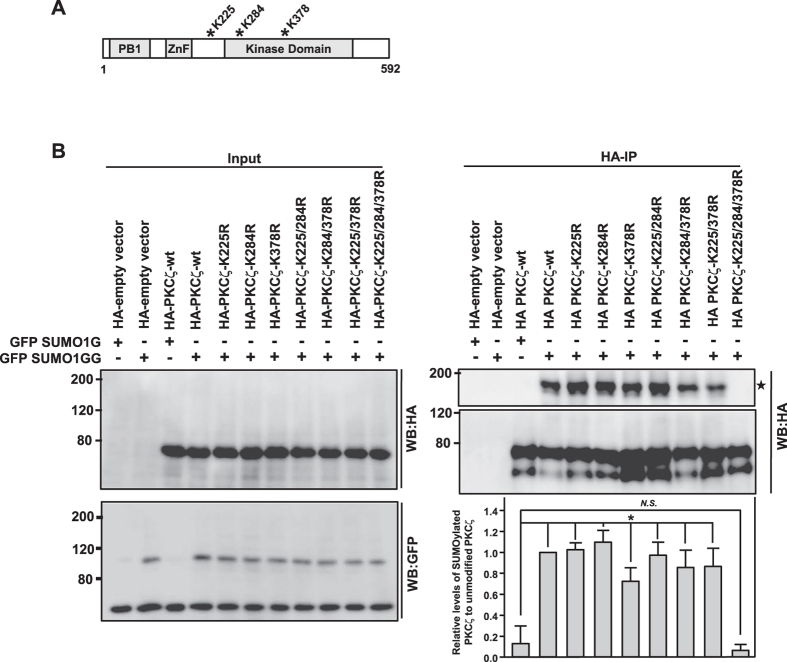
Identification of SUMOylation sites in PKCζ. (**A**) Schematic diagram of PKCζ depicting the domain architecture: PB1 (Phox and Bem 1), ZnF (zinc-finger) and kinase domains. Potential lysine (K) residues that were predicted by SUMOplot software as SUMOylation sites are marked with asterisks. (**B**) HEK293T cells were transfected with GFP-SUMO1G or GFP-SUMO1GG along with HA-empty vector control, HA-PKCζ-wt, HA-PKCζ lysine (K) to arginine (R) single mutants (K225R, K284R, K378R), double mutants (K225/284R, K284/378R, K378/225R) or triple mutant (K225/284/378R). Cells were lysed 36 h post-transfection and co-IP was performed using anti-HA antibodies, followed by western blot analysis with indicated antibodies. For better viewing of unmodified and SUMO-modified PKCζ, western blots corresponding to different exposures are provided (HA-IP and HA-WB panels). The graph represents quantitative data depicting levels of SUMOylated aPKC relative to unmodified PKCζ in the corresponding immunoprecipitates. Error bars indicate standard deviations, *n* = 3, *P* values were calculated by one-way ANOVA. *P* value is considered statistically significant if it is less than the critical level, and is denoted by *. *N.S.* indicates non-significant.

Covalent attachment of SUMO to the target protein occurs at specific lysine (K) residues, mostly within the consensus sequence ψKxE/D (ψ, a bulky aliphatic residue; K, lysine; x, any amino acid; E, glutamic acid; D, aspartic acid), by formation of an isopeptide bond between the target lysine and the carboxyl-terminal glycine residue of SUMO peptide^27^. To identify the lysine residue in PKCζ involved in SUMO conjugation, we searched for the presence of consensus SUMOylation sites in PKCζ using the SUMOplot^T^ software (Abgent- http://www.abgent.com/sumoplot). The analysis predicted three potential lysine residues in PKCζ (K225, K284 and K378) with relatively higher score (Fig. 2A). All the three K residues were mutated to arginine (R) individually and in combination. Immunoprecipitation assays suggested that when all the three lysine residues were mutated to arginine, SUMOylation of PKCζ was significantly reduced. All the single and double mutants were significantly SUMOylated as compared to control [PKCζ-wild type (wt) co-transfected with GFP-SUMO1G] (Fig. 2B). As the triple mutant (K225/284/378R) showed almost complete loss of SUMOylation, we refer to this as PKCζ-SUMOylation-defective mutant (PKCζ-Smut).

**Figure 3:**
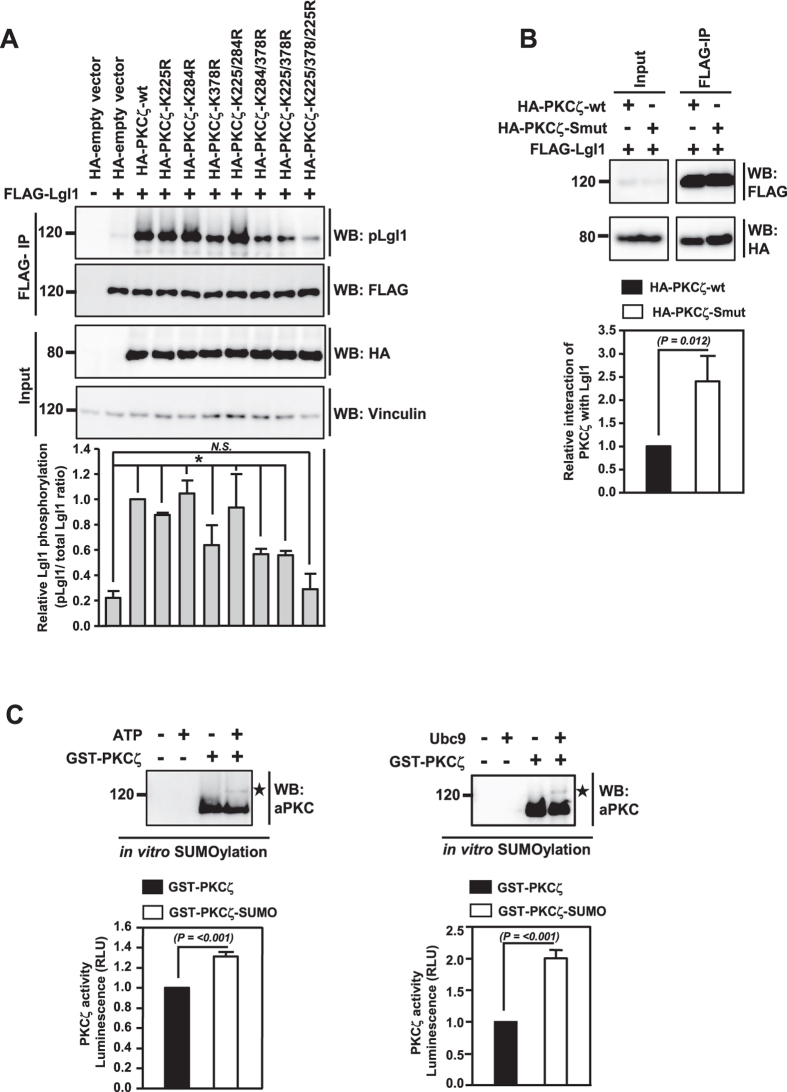
SUMOylation enhances aPKC activity. (**A**) HA-empty vector control, HA-PKCζ-wt, single HA-PKCζ lysine (K) to arginine (R) mutants (K225R, K284R, K378R), double mutants (K225/284R, K284/378R, K378/225R) or triple mutant (K225/284/378R) were co-expressed with FLAG-Lgl1 in HEK293T cells. Cells were lysed, Lgl1 was immunoprecipitated using FLAG-specific antibodies, and western analysis of the immunoprecipitates was performed with p-Lgl1/2, HA and FLAG antibodies. The graph represents quantitative data depicting levels of phosphoLgl1 relative to Lgl1 levels. Error bars indicate standard deviations, *n* = 3, *P* values were calculated by one-way ANOVA. *P* value is considered statistically significant if it is less than the critical level, and is denoted by *. *N.S.* indicates non-significant. (**B**) FLAG IP was performed in lysates prepared from HEK293T cells co-expressing HA-PKCζ-wt or HA-PKCζ-Smut with FLAG-Lgl1. The immunoprecipitates were subjected to WB with HA and FLAG antibodies. The graph represents quantitative data depicting relative interaction of PKCζ with Lgl1. Error bars indicate standard deviations, *n* = 3, *P* values calculated by Student’s *t* test. (**C**) *In vitro* kinase assay was performed using *in vitro* SUMOylated PKCζ. GST-PKCζ was subjected to *in vitro* SUMOylation reaction using recombinant proteins. The reactions were performed in the presence (+) or absence (−) of ATP (top left panel) and presence (+) or absence (−) of Ubc9 (top right panel). The reactions were subjected to immunoblotting using anti-aPKC antibodies. *indicates SUMOylated PKCζ. The PKCζ from *in vitro* SUMOylation reactions was further assayed for the activity using a commercial kit as per manufacturer’s instruction. The graph represents quantitative data showing relative activity of PKCζ derived from indicated reactions, expressed as relative luciferase units (RLU). Error bars indicate standard deviations, *n* = 3, *P* values calculated by Student’s *t* test.

Next, we wished to analyze the effect of SUMOylation on the activity of aPKC. As a critical player in many cellular processes, aPKC exerts its effect through phosphorylation of key proteins^8^. To test whether SUMOylation affects aPKC’s kinase activity, we monitored the phosphorylation status of one of its targets, Lgl. We co-expressed FLAG-tagged Lgl1 (one of the Lgl isoforms) with HA-PKCζ-wt, HA-PKCζ K to R single mutants (K225R, K284R, K378R), double mutants (K225/284R, K284/378R, K378/225R) or triple mutant (K225/284/378R or PKCζ-Smut), and analyzed the extent of Lgl1 phosphorylation using a phospho (p)-Lgl specific antibody. PKCζ-wt expression significantly enhanced Lgl1 phosphorylation, as compared to control vector transfected cells. PKCζ mutants K225R, K284R, and K225/284R also showed kinase activity comparable to that of the wild-type PKCζ. However, although PKCζ-K378R, K284/378R, K378/225R mutants had reduced activity as compared to that of PKCζ-wt, these mutants were still capable of phosphorylating Lgl to a significant level as compared to HA-vector control. Importantly, the PKCζ-Smut did not show any increased activity as compared to that of HA-control (Fig. 3A), indicating that SUMOylation enhances the aPKC activity *in vivo*. Furthermore, co-immunoprecipitation experiments suggested that PKCζ-Smut exhibited better interaction with Lgl1 as compared to PKCζ-wt (Fig. 3B). This is consistent with the previous finding that phosphorylation of Lgl compromises with its ability to bind to aPKC^13^,^14^,^15^. These experiments suggested that SUMOylation could increase the kinase activity of aPKC.

To confirm the SUMOylation-dependent activation of aPKC, recombinant GST-PKCζ was SUMOylated using *in vitro* SUMO1 conjugation system in the presence and absence of ATP and was then used for *in vitro* kinase assays. As expected, PKCζ was modified with SUMO only in the presence of ATP and this resulted in its enhanced activity (Fig. 3C, left panel). Only a small fraction of the total PKCζ was SUMOylated *in vitro* (~3%), which accounted for an increase of ~30% kinase activity. We also assayed the PKCζ activity after subjecting to *in vitro* SUMOylation in the absence and presence of Ubc9, and confirmed that SUMOylation has a specific role in activating aPKC. Under this condition, the fraction of PKCζ that was SUMOylated was ~10%, which accounted for an increase of ~100% kinase activity (Fig. 3C right panel). Collectively, these *in vitro* studies indicated that SUMOylation of PKCζ increased its activity approximately by 10-fold.

Phosphorylation of PKCζ at a specific threonine (T) residue in the activation loop (T410) and in the turn motif (T560) is important for its activation^9^,^10^. We sought to determine the phosphorylation status of SUMOylated PKCζ. For this, HEK293T cells were co-transfected with HA-PKCζ along with SUMO1G or SUMO1GG, and the immunoprecipitated HA-PKCζ was probed with phospho-T410 or phospho-T560 antibodies (Supplementary Figure 1). The results indicated that the SUMOylated form of PKCζ is phosphorylated at both the sites similar to the non-SUMOylated PKCζ.

**Figure 4:**
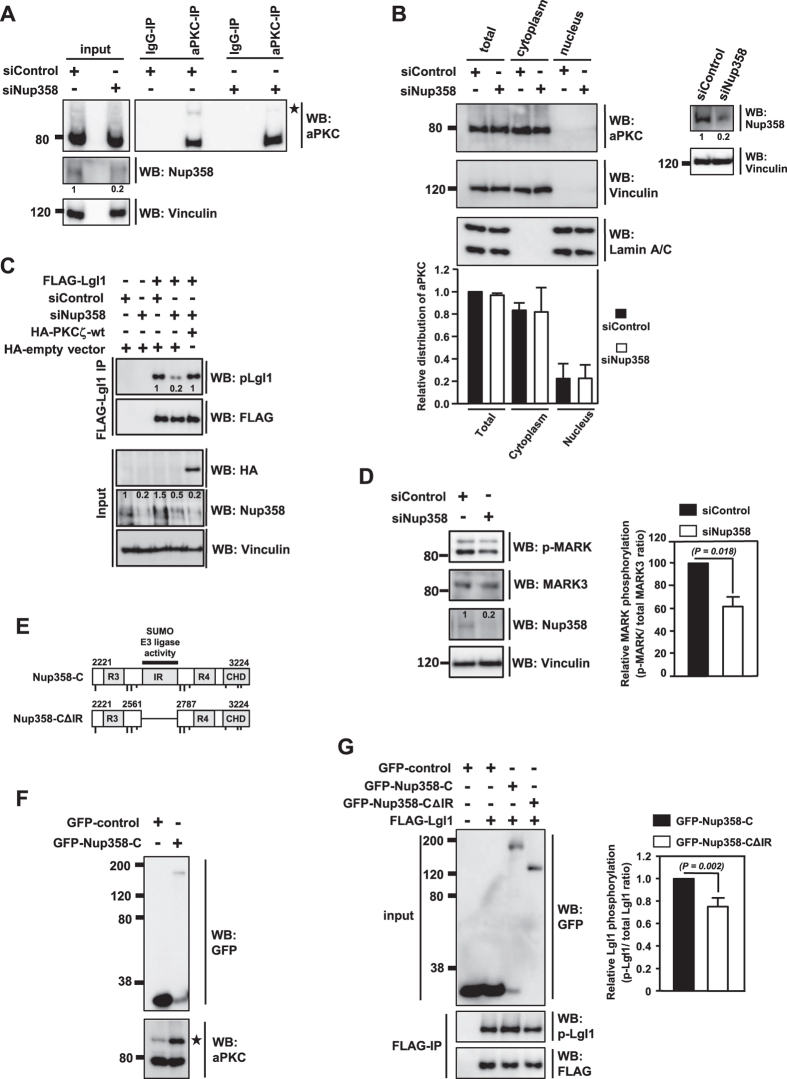
Nup358 acts as E3 ligase for aPKC SUMOylation. (**A**) HEK293T cells were transfected with control (siControl) or Nup358 specific (siNup358) siRNA and were lysed and analyzed for the levels of indicated proteins using specific antibodies by western blotting (WB). *indicates SUMO-modified band. Extent of Nup358 depletion was monitored with Nup358 specific antibodies. Vinculin was used as loading control. Numbers denote relative intensities of indicated bands. (**B**) siRNA treated cells were subjected to nucleo-cytoplasmic fractionation and presence of aPKC in the nuclear and cytoplasmic fractions was assessed by WB. Lamin A/C and Vinculin were used as markers for nuclear and cytoplasmic fractions, respectively. The graph represents quantitative data for relative aPKC nucleo-cytoplasmic distribution under control and Nup358 depleted conditions. Error bars indicate standard deviations, *n* = 3, *P* values calculated by Student’s *t* test. The extent of Nup358 depletion was monitored by western blotting with specific antibodies, and Vinculin was used as loading control. Numbers denote relative intensities of indicated bands. (**C**) Cells were initially transfected with siControl or siNup358 and were retransfected with FLAG-Lgl1 with (+) or without (−) HA-PKCζ. Cell lysates were analyzed by WB using indicated antibodies. Numbers denote relative intensities of indicated bands. (**D**) Lysates prepared from HEK293T cells transfected with siControl or siNup358 were analyzed for the levels of indicated proteins. Vinculin was used as loading control. Graphs represent quantitative data for relative levels of phosphorylated p-MARK2 as compared to total MARK3. Error bars indicate standard deviations, *n* = 3, *P* values calculated by Student’s *t* test. (**E**) Schematic depiction of C-terminal region of human Nup358 (Nup358-C) with amino acids marked in number. Nup358-C contains two RanGTP binding domians (RB3 and RB4), internal repeat (IR) that acts as SUMO E3 ligase and cyclophilin homology domain (CHD). Dashed line shows the deleted region in Nup358-C mutant (Nup358-CΔIR). (**F**) HEK293T cells were transfected with GFP-control or GFP-Nup358-C construct and monitored for SUMOylation of endogenous aPKC using specific antibodies by WB. *indicates SUMOylated aPKC species. (**G**) Cells were co-transfected with indicated constructs and lysates were immunoblotted for indicated proteins. Graph represents quantitative data depicting the relative p-Lgl1 levels compared to total Lgl1 levels. Error bars indicate standard deviations, *n* = 3, *P* values calculated by Student’s *t* test.

SUMO E3 ligases mediate the last step in SUMO conjugation, by transferring SUMO from E2 to the substrate. Earlier reports showed that Nup358, a well characterized SUMO E3 ligase, interacts with aPKC^17^. We wished to investigate if Nup358 functions as the E3 ligase for aPKC SUMOylation. Towards this, we analyzed the SUMOylation status of aPKC in Nup358 depleted HEK293T cells. Our results suggested that the extent of aPKC modification was significantly reduced in Nup358 depleted cells as compared to control siRNA treated cells (Fig. 4A).

Next, we tested if Nup358 knockdown caused any difference in the nucleo-cytoplasmic distribution of aPKC. The levels of aPKC in the nuclear and/or cytoplasmic fractions showed no detectable changes in the presence or absence of Nup358 (Fig. 4B), indicating that this nucleoporin does not play a major role in regulating the nucleo-cytoplasmic transport of aPKC. Furthermore, we also analyzed the intracellular localization of GFP-PKCζ-wt and GFP-PKCζ-Smut by fluorescence microscopy. The nucleo-cytoplasmic distribution of PKCζ-wt and PKCζ-Smut was comparable (Supplementary Figure 2A). Moreover, the overall localization pattern of GFP-PKCζ-wt remained unaffected in the absence and presence of endogenous Nup358 (Supplementary Figure 2B). Together, these results suggested that the decreased level of SUMOylated aPKC in Nup358 depleted condition was not majorly due to an indirect effect caused by difference in the nucleo-cytoplasmic distribution of aPKC.

Further, we monitored the activity of aPKC by assessing the phosphorylation status of its substrate, Lgl1, in Nup358 deficient cells. Our results showed that there was about 40–50% reduction in the p-Lgl1 levels in Nup358 knockdown cells as compared to control cells (Fig. 4C). Interestingly, ectopic expression of HA-PKCζ in Nup358 knockdown cells rescued the phosphorylation of Lgl1 to levels comparable to the control siRNA treated cells (Fig. 4C). Furthermore, we found that the phosphorylation of another aPKC substrate, a member of the partitioning-defective 1 (Par1)/microtubule affinity-regulating kinase (MARK) family, was also significantly reduced (~40%) in Nup358 depleted cells as compared to control cells (Fig. 4D). These results indicated that Nup358 is important for endogenous aPKC activation.

We wished to investigate if the activation of aPKC required Nup358’s E3 ligase function. Ectopic expression Nup358-C (Fig. 4E), a fragment of Nup358 having the E3 ligase activity, considerably increased the extent of endogenous aPKC SUMOylation in HEK293T cells (Fig. 4F). Moreover, expression of Nup358-C, but not an E3 ligase-defective mutant of Nup358-C (Nup358-CΔIR) (Fig. 4E), activated endogenous aPKC as monitored by the phosphorylation status of Lgl (Fig. 4G). Collectively, these results support the conclusion that Nup358 stimulates the aPKC activity by functioning as its SUMO E3 ligase.

## Discussion

The activity of aPKC is known to be controlled by different mechanisms^28^. Here we show that SUMO-modification of aPKC enhances its activity and the nucleoporin Nup358 acts as its SUMO E3 ligase. This additional level of aPKC regulation through SUMOylation could have implications in multiple aPKC-regulated processes. Our studies also indicated that Par6 might be regulated by SUMO conjugation. However, the effect of Par6 SUMOylation, if any, on the activation of aPKC in our experimental set up is not known. It is formally possible that SUMOylation of other aPKC interactors also could indirectly contribute to the activation of aPKC *in vivo*. Nevertheless, our data on the activation of PKCζ through *in vitro* SUMOylation, supports a direct role for SUMOylation in the regulation of aPKC activity *in vivo*.

Members of aPKC have a conserved role in cell polarity across multicellular organisms ranging from *Caenorhabditis elegans* to humans and in multiple cell types^29^. Previous studies from our laboratory had suggested a role for Nup358 in the regulation of cell polarity during directed migration and neuronal differentiation^23^,^30^. Localization of the microtubule plus end binding protein Adenomatous polyposis coli (APC) to the leading edge has been shown to regulate polarized cell migration^31^. The microtubule association and cortical localization of APC is regulated by aPKC-dependent inhibition of GSK3β, which otherwise phosphorylates and inhibits the binding of APC to microtubules^32^. As our previous studies had shown that Nup358 interacts with APC and regulates its localization to the leading edge^30^, the data presented here raises the possibility that Nup358’s role in the regulation of APC localization could involve activation of aPKC through SUMOylation.

Our data indicate that Nup358 plays a role in regulating the phosphorylation and activity of Lgl through aPKC SUMOylation. Spatiotemporal regulation of Lgl is important for the establishment and maintenance of polarity in different cellular contexts such as neuronal differentiation, epithelia formation and directed cell migration^29^. This raises the possibility that Nup358 may function in the process of polarity through the modulation of Lgl activity. Future investigation will be required to explore these possibilities.

Previous studies have shown that Nup358 is not absolutely essential for general import and export of cargos in and out of the nucleus^19^,^33^. However, there appears to be a role for Nup358 in the nucleo-cytoplasmic transport of specific molecules in a cargo- and receptor-dependent manner^34^,^35^,^36^,^37^,^38^. In the present study, although we did not observe any discernible difference in the nucleo-cytoplasmic distribution of aPKC between control and Nup358 depleted conditions, we cannot completely rule out any transport function of Nup358 indirectly contributing to the regulation of aPKC activity or Lgl phosphorylation *in vivo*. However, as the conclusion that Nup358 regulates aPKC activity by SUMOylation is supported by multiple experiments in the present study, we consider the indirect effect to be unlikely.

Recently, it was shown that PKCα gets SUMOylated, which inhibits its activity^39^. Additionally, SUMOylation also regulates the function of PKCθ in T cell synapse organization and activation^40^. Based on our studies, aPKC gets added to the growing list of PKC family members being regulated by SUMOylation. The functional outcome of Nup358-mediated SUMOylation and activation of aPKC in multiple cellular processes awaits further research.

## Experimental Procedures

### Cell lines and Transfections

HEK293T and HeLa S3 cell lines were cultured in Dulbecco’s Modified Eagle’s Medium (Invitrogen) supplemented with 10% (v/v) fetal bovine serum (Invitrogen) and 1% antibiotics (Ciprofloxacin) in a humidified incubator at 37 °C under 5% CO_2_ condition. The cells were grown in 100 mm petri-plate (1 × 10^7^ cells per plate) or in six-well plate (3 × 10^5^ cells per well) for immunoprecipitation and western analysis. After 12 h, cells were transfected using polyethylene imine linear (PEI, MW-25000 kDa, Polysciences Corporation Ltd.) following manufacturer’s instructions.

### siRNA

For siRNA transfections, Lipofectamine RNAiMax (Invitrogen) was used according to manufacturer’s instructions. All siRNAs were used at a final concentration of 40 nM. The siRNAs were synthesized (Dharmacon) against the following target sequences: siNup358 (5′ GGACAGTGGGATTGTAGTG 3′)^41^, siControl (5′ TTCTCCGAACGTGTCACGT 3′)^41^.

### Plasmids

For generation of pEGFP-C1-SUMO1G, pEGFP-C1-SUMO1GG, pEGFP-C1-SUMO2G and pEGFP-C1-SUMO2GG, the ORF was PCR amplified from pCMV-Myc-SUMO1 or pCMV-Myc-SUMO2 (kind gifts from Michel Goossens, INSERM, France) as the templates with appropriate primers and cloned at KpnI-SmaI sites of pEGFP-C1 (Clontech). The pCAN-HA-hPKCζ (HA-PKCζ) was a gift from Steven Martin (University of California, Berkeley, USA), which was used for generating the following mutants by PCR based method; pcDNA-HA-hPKCζ-K225R, pcDNA-HA-hPKCζ-K284R, pcDNA-HA-hPKCζ-K378R, pcDNA-HA-hPKCζ-K225/284R, pcDNA-HA-hPKCζ-K225/378R, pcDNA-HA-hPKCζ-K284/378R, pcDNA-HA-hPKCζ-K225/284/378R (also called hPKCζ-Smut), pEGFP-hPKCζ-wt, pEGFP-hPKCζ-Smut. pCAN-Myc-hPar6C (Myc-Par6) was kindly provided by Steven Martin (University of California, Berkeley, USA). The rat Par3 homolog ASIP was subcloned from SRHisB-rASIP (a kind gift from Shigeo Ohno, Yokohama City University School of Medicine, Japan) into pcDNAHA-C1 at the EcoRV-XhoI to generate pcDNA-HA-Par3 (HA-Par3). pFlagCMV2-mLgl1 was generously provided by Tony Pawson (Mount Sinai Hospital, Canada). pEGFP-Nup358-C has been described previously^42^. pEGFP-Nup358-CΔIR was generated by deleting SpeI-KpnI fragment encompassing amino acids 2562 to 2786 of Nup358. Detailed cloning information is available upon request.

### Immunoprecipitation and western blotting

Cells were washed with ice-cold TBS, collected using a cell scrapper and resuspended in chilled NP40 lysis buffer (20 mM Tris-HCl, pH 8, 137 mM NaCl, 10% glycerol, 2 mM ethylenediaminetetraaceticacid (EDTA), 20 mM N-ethylmaleimide (NEM) and 1% NP40). Cells were lysed by sonicating at 4 °C with 30% amplitude (1 sec ON and 2 sec OFF for 5 times) at an interval of 15 minutes thrice using VibraCell (Sonics & Materials, Inc.). Samples were centrifuged at 12,000 rpm for 30 minutes at 4 °C in Eppendorf centrifuge (5417 R). The supernatant was used for western blotting (WB) and immunoprecipitation (IP) assays. Protein concentration was measured using Bradford assay (Bio-Rad).

For IP, indicated antibody was bound to 20 μl of Protein A Sepharose beads (Invitrogen) at 4 °C for 1 h by mixing on Rotospin Rotary Mixer (Tarson Products Pvt. Ltd.). Alternatively, for FLAG or HA IP, 20 μl of EZview™ Red anti-FLAG M2 Affinity Gel (Sigma-Aldrich) or anti-HA Affinity Gel (Sigma-Aldrich) was used. Then the beads were washed with TBS once. HEK293T cells were lysed in NP40 buffer and the lysate was incubated with antibody-bound beads for 1–2 h at 4 °C. The samples were centrifuged at 5,000 rpm for 5 min in Eppendorf centrifuge (5417 R), followed by one wash with the lysis buffer and two washes with TBS. For control IP, the same procedure was followed, except that instead of specific antibody, rabbit or mouse IgG (Vector Laboratories) was used. Then the bound proteins were directly eluted using 3x SDS loading dye. The eluted proteins were separated on SDS-PAGE, and then transferred to PVDF membrane using a semi-dry transfer apparatus (Bio-Rad or GE Healthcare). The membranes were probed with indicated antibodies diluted in the blocking buffer [0.5% BSA in TBS with 0.1% Tween-20 (TBS-T)] for 2 h at room temperature or at 4 °C overnight. After washing with TBS-T thrice, the membrane was incubated with HRP conjugated secondary antibody diluted in blocking buffer for 1 h at room temperature. Detection was performed using enhanced chemiluminescence substrate (ECL Plus, ThermoFisher Scientific). Images were captured using ImageQuant LAS 4000 (GE Healthcare). All the densitometric analyses of western blots were performed using Multi Gauge V3.0 software.

The following antibodies and dilutions were used for western blotting: mouse anti-Nup358 (1:3,000, Santa Cruz Biotechnology, sc-74518), mouse anti-GFP (1:6,000, Santa Cruz Biotechnology, sc-9996), mouse anti-aPKC (1:6,000, Santa Cruz Biotechnology, sc-17781), rabbit anti-aPKC (1:6,000, Santa Cruz Biotechnology, sc-216),. mouse anti-c-Myc (1:1,000, Santa Cruz Biotechnology, sc-40), mouse anti-Lamin A/C (1:3,000, Santa Cruz Biotechnology, sc-7292), mouse anti-Vinculin (1:10,000, Sigma, V9131), mouse anti-α-tubulin (1:10,000, Sigma, T5168), mouse anti-HA (1:1,000, Covance Research Products, MMS-101R), donkey anti-rabbit IgG-HRP (1:10,000, GE Healthcare, NA-934), sheep anti-mouse IgG-HRP (1:10,000, GE Healthcare NA-931), rabbit Anti-LLGL1 (phospho S655 + S659) + LLGL2 (phospho S645 + S649) (1:3,000, Abcam, ab59950), rabbit anti-PKCζ (1:3000, Cell Signaling Technology, #9368), rabbit anti-PKCζ (phospho T560) (1:3,000, Abcam ab62372), rabbit anti-PKCζ (phospho T410) (1:3,000, Santa Cruz Biotechnology sc-12894-R), rabbit anti-SUMO-1 (1:1,000, Cell Signaling Technology, #4930), rabbit anti-SUMO-2/3 (1:1,000, Cell Signaling Technology, #4971), rabbit anti-Ubc9 (1:3,000, Cell Signaling Technology, #4786S) mouse anti-Par1/MARK (1: 1,000, C-TAK1, EMD Millipore, 05–680) and rabbit anti-Par1/MARK2 (phospho T595) (1: 3,000, Abcam, ab34751).

### Immunofluorescence microscopy

HeLa S3 cells grown on glass coverslips were transfected with indicated constructs, fixed using chilled methanol for 5 min and washed thrice with TBS. Cells were then incubated with indicated primary antibodies diluted in TBS containing 2% normal horse serum (Vector Laboratories) for 90 min at room temperature (RT). Cells were then washed thrice with TBS, followed by addition of fluorescently conjugated secondary antibodies (Invitrogen) and incubation for 45 min at RT. Hoechst-33342 dye (Sigma) was used to stain the DNA, which was added to the secondary antibody solution. Cells were again washed thrice with TBS and the coverslips were mounted on glass slides using Vectashield mounting medium (Vector Laboratories). To avoid dehydration, coverslips were sealed using nail polish, and were later observed and images were captured with fluorescence microscope (Leica TCS SP5). Images were processed using Adobe Photoshop CS2. Rabbit anti-Nup358 (1:1,000) generated in the lab^41^ was used for immunofluorescence studies.

### *In vitro* SUMOylation and Kinase assay

100 ng of GST-PKCζ (Promega) was conjugated with SUMO1 at 37 °C for 2 h using *in vitro* SUMOylation kit (Boston Biochem) as per manufacturer’s instructions. The extent of SUMOylation was assessed by western blotting. After *in vitro* SUMOylation, 1 ng of GST-PKCζ or SUMO modified GST-PKCζ was used in *in vitro* kinase assay, which was performed at 30 °C for 1 h using ADP-Glo^TM^ Kinase assay kit (Promega) as per manufacturer’s protocol. The luminescence was recorded using GloMax Multi Detection System (Promega).

### Nucleo-cytoplasmic fractionation

Control or Nup358 siRNA transfected HEK293T cells were lysed in cytoplasmic extraction buffer (20 mM HEPES, pH8.0, 10 mM KCl, 1 mM MgCl_2_, 20% glycerol, 1% Triton X-100). Lysis was done at 4 °C by gently pipetting the lysate up and down thrice at an interval of 10 min. The lysate was centrifuged in Eppendorf centrifuge (5417 R) at 12,000 rpm for 15 min. Cytoplasmic fraction was collected and the pellet was washed three times with TBS. Further, pellet was lysed in Nuclear lysis buffer (20 mM HEPES, pH 7.4, 150 mM NaCl, 1.5 mM MgCl_2_, 2 mM EGTA, 2 mM DTT, 1% Triton X-100). Lysate was then centrifuged in Eppendorf centrifuge (5417 R) at 12,000 rpm at 4 °C for 20 min, and the supernatant (nuclear fraction) was collected. Total protein fraction was prepared by mixing equal proportion of cytoplasmic and nuclear lysates, and analysis of total protein fraction was done by loading double the volume of total protein lysate. The proteins were resolved on SDS-PAGE and analyzed by western blotting. Vinculin and LaminA/C were used as markers for cytoplasmic and nuclear fractions, respectively.

### Statistical Analysis

The experiments were independently repeated at least three times, and the values are expressed as mean ± SD. *P* values were calculated using Student’s *t* test (SigmaStat 3.5). *P* value ≤ 0.05 was considered statistically significant. Graphs were plotted using (SigmaPlot 10.0). For Figs 2B and 3A, one-way ANOVA was performed and *P* values less than the critical level was considered statistically significant.

## Additional Information

**How to cite this article**: Yadav, S. K. *et al.* Regulation of aPKC activity by Nup358 dependent SUMO modification. *Sci. Rep.* **6**, 34100; doi: 10.1038/srep34100 (2016).

## Supplementary Material

Supplementary Information
