# Supplementary material for: Regulation of aPKC activity by Nup358 dependent SUMO modification
Source: Sci Rep. 2016 Sep 29;6:34100. doi: 10.1038/srep34100 (PMC5040961; doi:10.1038/srep34100)
Supplement: Supplementary Information [file srep34100-s1.pdf]

Supplementary Information

Regulation of aPKC activity by Nup358 dependent SUMO modification

Santosh Kumar Yadav, Indrasen Magre, Aditi Singh, Deepak Khuperkar and Jomon Joseph*

National Centre for Cell Science, S.P. Pune University Campus, Ganeshkhind, Pune 411007

*** Correspondence: Email: josephj@nccs.res.in**

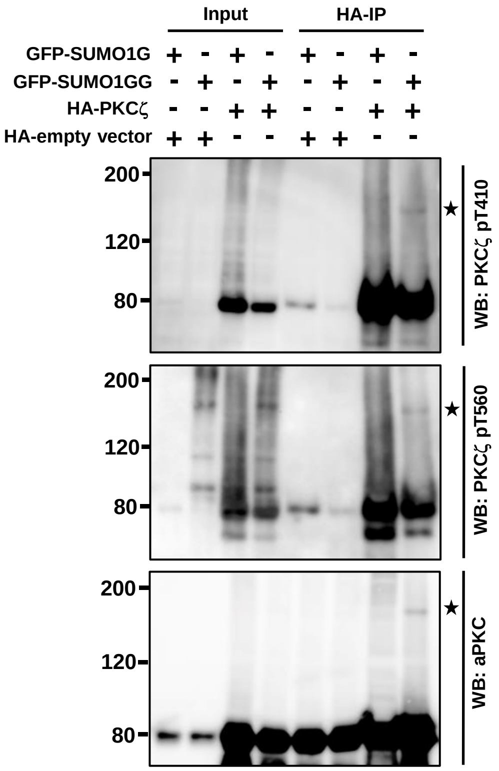

Supplementary Figure 1. Phosphorylation status of the SUMOylated PKCζ. (A) HEK293T cells were transfected with GFP-SUMO1G or GFP-SUMO1GG along with HA-empty vector control or HA-PKCζ-wt. Cells were lysed 36 h post-transfection and co-immunoprecipitation (IP) was performed using anti-HA antibodies. The immunoprecipitates were analyzed by western blotting (WB) with specific antibodies that detect aPKC (bottom most panel), and PKCζ phosphorylated at T410 (pT410, middle panel) or at 560 (pT560, middle panel). * indicates SUMOylated PKCζ.

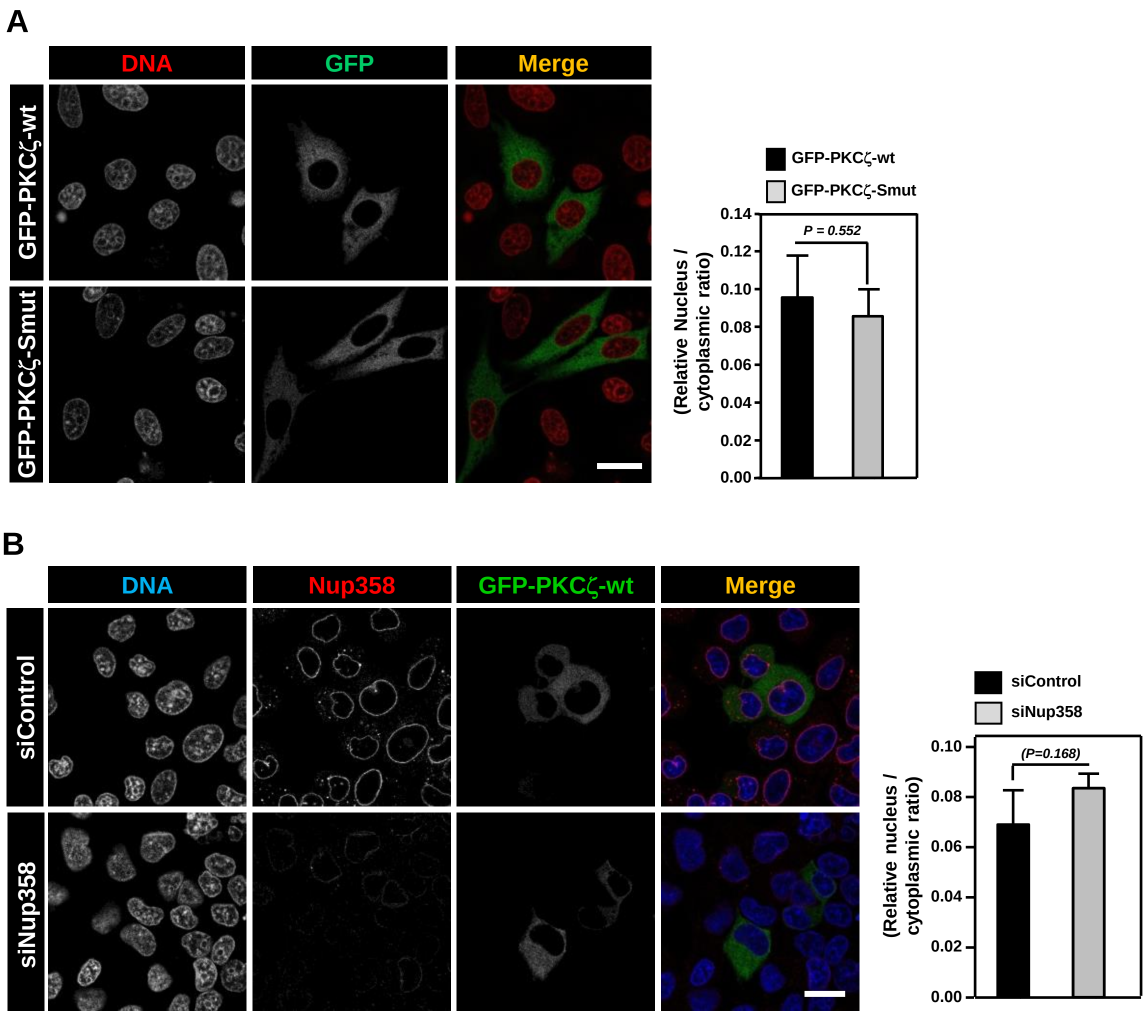

Supplementary Figure 2. SUMOylation does not affect the nucleo-cytoplasmic distribution of PKCζ. (A) HeLa cells were transfected with GFP-PKCζ-wt or GFP-PKCζ-Smut construct. Cells were fixed and ectopically expressed GFP-PKCζ-wt or GFP-PKCζ-Smut (green) was detected by epifluorescence. DNA was stained with Hoechst 33342 (pseudocoloured in red). Scale bars, 20 μm. The graph represents the relative nucleus / cytoplasmic distribution of GFP-PKCζ-wt or -Smut, calculated by analyzing the fluorescence intensities between nuclei and cytoplasm of 10 cells from each of 3 independent experiments Error bars indicate standard deviations, n = 3, P values calculated by Student's t test. (*B*) HeLa cells were initially transfected with control (siControl) or Nup358 specific (siNup358) siRNA and were later retransfected with GFP-PKCζ-wt construct. Cells were fixed and stained for Nup358 (red) using a specific antibody, and GFP-PKCζ-wt (green) was detected by epifluorescence. DNA was stained with Hoechst 33342 (blue). Scale bars, 20 µm. The graph represents the relative nucleus / cytoplasmic distribution of GFP-PKCζ, calculated by analyzing the fluorescence intensities between nuclei and cytoplasm of 10 cells from each of 3 independent experiments Error bars indicate standard deviations, n = 3, P values calculated by Student's t test.
